# Molecular Analytical Assessment of Thermally Precipitated α-Lactalbumin after Resolubilization

**DOI:** 10.3390/foods10092231

**Published:** 2021-09-20

**Authors:** Nicole Haller, Isabel Maier, Ulrich Kulozik

**Affiliations:** Chair of Food and Bioprocess Engineering, TUM School of Life Sciences, Technical University Munich, Weihenstephaner Berg 1, 85354 Freising, Germany; isabel.muthmann@tum.de (I.M.); ulrich.kulozik@tum.de (U.K.)

**Keywords:** α-lactalbumin, refolding, resolubilization, whey protein, thermal denaturation, native, FTIR, DSC

## Abstract

Selective thermal precipitation followed by a mechanical separation step is a well described method for fractionation of the main whey proteins, α-lactalbumin (α-la) and β-lactoglobulin (β-lg). By choosing appropriate environmental conditions the thermal precipitation of either α-la or β-lg can be induced. Whereas β-lg irreversibly aggregates, the precipitated α-la can be resolubilized by a subsequent adjustment of the solution’s pH and the ionic composition. This study reports on the analytical characterization of resolubilized α-la compared to its native counterpart as a reference in order to assess whether the resolubilized α-la can be considered close to ‘native’. Turbidity and quantification by RP-HPLC of the resolubilized α-la solutions were used as a measure of solubility in aqueous environment. RP-HPLC was also applied to determine the elution time as a measure for protein’s hydrophobicity. DSC measurement was performed to determine the denaturation peak temperature of resolubilized α-la. FTIR spectroscopy provided insights in the secondary structure. The refolding of α-la achieved best results using pH 8.0 and a 3-fold stoichiometric amount of Ca^2+^ per α-la molecule. The results showed that the mechanism of aggregation induced by gentle thermal treatment under acidic conditions with subsequent mechanical separation is reversible to a certain extent, however, the exact native conformation was not restored.

## 1. Introduction

Whey is a source of protein with excellent techno-functional properties and bioactive peptides. The key components, β-lactoglobulin (β-lg) and α-lactalbumin (α-la), make up for approximately 50% and 20% of the whey protein content, respectively. Due to their individual functional or physiological properties, the isolation in pure fraction is of increasing interest. Since several years, the development of cost-efficient separation methods of the individual whey protein fractions has evolved as an important driver for innovation in dairy industry and food research. A common concomitant of traditional dairy processing is the partial loss of native conformation caused by thermal, mechanical or chemical stresses on the protein [1].

α-la incorporates specific physiological and medical functionalities and a nutritional value that makes it an interesting candidate for the use as food additive or as a part of therapeutic concepts. Bovine α-la’s richness in tryptophan and its similarity to the human α-la makes it an ideal source for infant food formulations, allowing further adaption to the golden standard, mother’s milk [2,3]. Of special interest is its property to carry different minerals, such as Mg^2+^, Mn^2+^, Na^+^, K^+^, and of course Ca^2+^, which is natively bound in the holo-state [4]. α-la is reported to enhance the regeneration of cells [5], to possess antimicrobial properties against a broad spectrum of bacteria, including antibiotic resistant strains [6,7], and to have antitumor properties in a complex with oleic acid [8,9].

Generally, there are two ways for the separation of the whey protein main components that are based on selective aggregation of one of the fractions. An aggregation of β-lg can be initiated by a short heat treatment at high temperatures, which is irreversible due to the covalent disulfide bridge formation. The alternative is to selectively precipitate α-la under acidic conditions, which are not affecting β-lg. In both cases the aggregated fraction can easily be separated either by microfiltration or by centrifugation in order to obtain the native other fractions in the supernatant or permeate, respectively. The difference between these concepts is that in the second route, the aggregated α-la can be resolubilized, as shown by Toro-Sierra et al. [10], and both fractions were finally be assumed to be obtained in their native soluble states.

In any case, it should be priority to keep the nativity of the proteins at a maximum throughout the processing. Primarily, the bioactivity and anti-inflammatory or immunomodulating properties are substantially dependent on the native state of the whey proteins [11,12,13]. From a nutritional perspective, it is reported that whey proteins in their native states promote gastro-intestinal tolerance and maturation of infants [14,15]. Furthermore, Abbring et al. [16,17] showed that native whey protein has a lower allergenicity than the processed, i.e., heat-treated, equivalent. Additionally, any further processing will benefit from using standardized raw material with defined properties rather than a coincidental product with fluctuating characteristics.

In this study, the method of acidic precipitation of α-la with gentle heat treatment and subsequent separation from soluble and native β-lg was used. A previous study demonstrated that this process in combination with a separation by a decanter centrifuge provides high yields, easy scalability, and high purities of the obtained fractions [18]. With a comparably low heat load and a good stability at acidic pH values, the nativity of β-lg remains virtually unaffected by this method as demonstrated by Kella and Kinsella [19].

Back in 1964, Kronman et al. [20] reported on the release of the bound Ca^2+^-ion from α-la at an acidic pH, followed by its thermally induced aggregation. An additional calcium-complexing agent, such as EDTA [21], sodium hexametaphosphate [22], lactic acid [23], or citrate [24] prevents the re-transition to holo-state. In the calcium-free apo-form, the hydrophobic parts of the α-la protein are easier accessible, which is further promoted by gentle heating up to 60 °C [25]. Under these conditions, α-la forms precipitates, most likely caused by a combination of hydrophobic and electrostatic interactions [26]. The formed precipitates enable a size-based separation either by microfiltration [10,27] or centrifugation [18,28,29].

Already in the early stages of research in this field, the possibility to resolubilize the α-la precipitate by increasing the pH plus the addition of calcium was reported [30,31]. In fact, several studies proved that α-la is able to refold from a thermally unfolded apo-state to a native holo-conformation [4,6,21]. However, all of these studies have been performed under ideal and protected laboratory conditions, excluding any additional mechanical stress. It is highly questionable if the results of these isolated folding studies are transferable to industrial-like whey protein processing.

A definite proof is still lacking, whether α-la returns to the same globular folded state after the acidic precipitation and mechanical separation compared to the native holo-state. Therefore, the aim of this study was to characterize the molecular state of α-la after selective precipitation, separation and subsequent resolubilization. Turbidity by means of optical density and quantification by RP-HPLC were used as a measure for dissociation of precipitates upon basification. Differential scanning calorimetry (DSC) was used to determine the denaturation peak temperature of refolded α-la and to compare its thermal stability with its native reference. The Amid I band was analyzed by Fourier-transform infrared spectroscopy (FTIR), which allows quantification of certain secondary structure motifs such as α-helix, β-sheet, and β-turns [32]. The aim was to answer the question whether or to which extent the original ‘nativity’ of α-la—after having been exposed to thermal, chemical and mechanical stress—can be re-established.

## 2. Materials and Methods

### 2.1. Materials

Whey protein isolate (WPI) from Davisco (Le Seur, MN, USA) was used as raw material to produce the α-la precipitate suspension. This WPI has a protein content of 94.2% per dry matter. Trisodium citrate dihydrate and citric acid monohydrate were purchased from Bernd Kraft GmbH (Duisburg, Germany). HCl, NaOH, CaCl_2_ and EtOH were purchased from Sigma Aldrich (Steinheim, Germany). Analytical grade trifluoroacetic acid (TFA) and acetonitrile for HPLC analysis were purchased from Sigma Aldrich (Steinheim, Germany). De-ionized water (DIW) was used for solution preparation and analytical methods. For the HPLC methods Milli-Q (Merck KGaA, Darmstadt, Germany) purified water was used.

### 2.2. α-La Precipitation, Separation, and Refolding Procedure

WPI powder was dissolved in DIW to a final protein concentration of 150 g/L and was gently stirred using a three-blade agitator at 350 rpm (Heidolph Elektro GmbH & Co. KG, Schwabach, Germany). Total mixing time was 12 h at a temperature of 4 °C. The pH value of the protein solution was adjusted to pH 3.4 using trisodium citrate and citric acid. A final citrate content of 60 g/L was achieved. The beaker glass with the protein solution was placed in a water bath, while it was gently mixed by a magnetic stirrer. Upon reaching the solution target temperature of 50 °C, the temperature was kept at 50 ± 1 °C for 120 min, followed by a fast cooling phase in ice water to stop precipitation process. The suspension was transferred to 50 mL tubes and centrifuged at 6000× *g* for 30 min (Multifuge 1 S-R, Heraeus Holding GmbH, Hanau, Germany) to separate precipitates from β-lg enriched supernatant. In a previous study, it was shown that a considerable amount of native β-lg remains in the sediment phase [18]. Two washing steps of the sediment using DIW with adjusted pH to 3.4 using 0.1 M HCl were performed to remove β-lg residues from the sediment. The washing procedure resulted in a sediment consisting of more than 99% α-la, based on RP-HPLC analysis. For the refolding, the sediment was diluted in a ratio of 1:5 in DIW and mixed for 30 min on a magnetic stirrer at 150 rpm. The pH was increased with 0.5 M and 0.1 M NaOH to adjust the pH value to the respective target value between pH 6.0–10.0. Subsequently, CaCl_2_ was added to a final stochiometric ratio of 3 ionic calcium ions per α-la molecule. The solution was stirred for another 30 min at room temperature and target pH was confirmed once more, before HPLC, FTIR and DSC measurements were performed.

### 2.3. Determination of Resolubilization Degree

After refolding of the α-la samples at different pH values and CaCl_2_ addition, the optical density at 550 nm (OD_550_) as a measure of turbidity was determined by a spectrophotometer (Ultrospec III, Pharmacia AG, Uppsala, Sweden) as described in [33]. Cuvettes with a path length of 1 cm were used. DIW served as reference.

The quantification as well as determination of the degree of resolubilization of α-la was performed by RP-HPLC analysis as described by in [18]. For calculation of resolubilization degree *RD* (Equation (1)), it was required to prepare one sample that was clarified from insoluble protein by a pH 4.6 isoelectric precipitation and centrifugation (*c_α-la, soluble_*), and another one that was left untreated and in original composition (*c_α-la, total_*). Both samples were pre-diluted with DIW, followed by dissolving 200 µL of properly homogenized sample in 800 µL of a 6 N guanidine buffer. The incubation time was at least 30 min. Afterwards samples were transferred to screw-capped glass vials and measured by RP-HPLC (Agilent 1100 Series chromatograph, Agilent Technologies, Waldbronn, Germany, equipped with Agilent Zorbax 300SB-C18, 4.6 × 150 mm, 5 µm).

(1) $$RD=c_{\alpha -la,\;\;soluble}/c_{\alpha -la,\;total}\times 100\%$$

### 2.4. Production of Native α-La as Analytical Standard

The reference standard for native α-la was manufactured in-house using the same starting material as for the precipitate production. The method of selective thermal aggregation of β-lg was used as described in [34]. In brief, a WPI solution with 2.5% protein content, pH of 7.5 and 0.5 g/L CaCl_2_ was heated at 97 °C for 9 s. Upon β-lg aggregation, pH was adjusted to 4.6 for isoelectric precipitation of denatured proteins and higher separation efficiency in centrifugal separation. The α-la-enriched supernatant was separated from the sediment containing denatured protein by a decanter centrifuge (MD 80, Lemitec, Berlin, Germany) at 4000× *g* and a throughput of 10 L/h. Subsequently, the pH was adjusted to 8.0 in order to increase α-la stability. A 10 kD cassette membrane filter cut-off was used for a 5-fold concentration of the solution and subsequent diafiltration steps using DIW to remove lactose and salts. The α-la standard was determined to have a nativity of 97%, containing 89% α-la, based on RP-HPLC analysis.

### 2.5. Characterization of Elution Time by RP-HPLC Analysis

Using a standardized and highly reproducible RP-HPLC analysis method for one specific protein allows to use the retention time as a measure for the proteins’ relative hydrophobicity.

The resolubilized α-la samples were diluted to a protein concentration of approximately 1%. The pH value was adjusted to 4.6 and the samples were filtered through a 0.45 µm syringe filter into a glass vial, before being analyzed be RP-HPLC. An Agilent 1100 chromatograph with a PLRP-S 8 µ 300 Å 150 × 4.6 mm column (Latek, Eppelheim, Germany) was used. Eluent A (1% TFA in water) and a gradient of eluent B (80% acetonitrile and 0.055% TFA in water) were used at a flow rate of 1 mL/min at 40 °C. The eluent was detected using an UV detector at 226 nm.

In the resulting chromatograms, the time point when the peak maximum of each α-la sample was reached was defined as the elution time. The elution times of the different α-la batches, which were resolubilized at different pH values between 6.5 and 9.5, were compared.

### 2.6. Determination of Denaturation Temperature by DSC

Unfolding temperature of the α-la samples was determined by DSC including the Refrigerated Cooling System (Q1000, TA Instruments, Alzenau, Germany). An amount of 20 µg of the refolded α-la solutions with an approximate protein content of 5% *w*/*w* were used as sample, while DIW was used as reference. For each measurement, one sample and a reference were given in aluminum pans and crimped with a lid, respectively. They were placed on the respective platforms in the measurement cell, which are equipped with heat flow sensors. The cell was closed, evacuated and the temperature was controlled as defined in the recipe. The first step was an equilibration at 10 °C for 5 min, followed by heating rate of 3 °C/min to 85 °C, which was held for 1 min. The final step was to ramp down to 10 °C with 10 °C/min. Conformational changes such as unfolding of the protein result in a deviating heat flow of the sample compared to the reference. Obtained data were evaluated by OriginPro 2020 to determine the heat flow peak maximum, which was defined as denaturation temperature of the sample.

### 2.7. Analysis of the Secondary Structure by FTIR

α-la secondary structure and success of refolding were analyzed by FTIR spectroscopy (Tensor 27, Bruker Optik GmbH, Ettlingen, Germany) equipped with an attenuated total reflectance (ATR) crystal. Before each measurement, the crystal was cleaned with DIW and 70% EtOH, and a background measurement with air was performed. Then, 5 µL of the sample was applied on the crystal and dried under regulated conditions (gaseous N_2_ overflow at flow rate of 100 mL/min for 10 min). Each measurement comprised 60 scans and each sample was applied and measured 5 times. The scans recorded the spectrum between 400–4000 cm^−1^ with a nominal instrument resolution of 2 cm^−1^.

Data evaluation was performed with the software OPUS 7.2.139.1294 (Bruker Optik GmbH, Ettlingen, Germany). Data preparation covered the following steps: The spectra were cut between 1600 and 1700 cm^−1^, which represents the so-called Amid I band that reflects protein secondary structures. Elastic baseline correction using 64 points was applied. Spectra were normalized by Min.-/Max.-method and afterwards second derivation of spectra were calculated. All obtained minima in second-derivative spectra indicate an inflection point in the original absorbance spectra, meaning each minimum represents a peak position at a certain wavenumber. A median spectrum was calculated from all appropriate spectra. Finally, Lorenz-deconvolution with noise reduction of 0.55 and band Shape 2 was applied. The Levenberg–Marquardt–Algorithm was used to calculate peak areas at the positions, previously determined by the second derivative. The integrated areas of each peak were related to secondary structural motives as described in [35].

### 2.8. Data Evaluation and Statistics

Presented data points are the mean values with standard deviations. The number of replications *n* is given in the respective figure description. Data were evaluated and plotted using OriginPro 2020 (OriginLab Corporation, Northampton, MA, USA). The *t*-test was applied to compare significance of difference between the reference and the refolded α-la samples at a confidence level of 95%. The Student’s *t*-test was used when the assumptions of normality and homogeneity of variances was fulfilled. In case of violation of the assumption of normality, Welch’s *t*-test was used. Statistical significance was declared at *p* < 0.05. Pairwise comparison between different refolding pH values was performed using one-way ANOVA and Tukey’s test as post-hoc test.

## 3. Results and Discussion

### 3.1. Solubilization of α-La Precipitates

**Figure 1:**
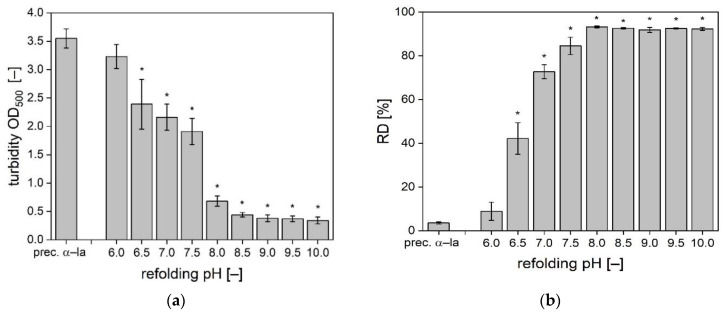
(**a**) OD_550_ as measure for turbidity of the precipitated and washed α-la and of resolubilized α-la samples after pH adjustment and CaCl_2_ addition. Data are given as mean ± SD, *n* = 3. * Significantly different than precipitated α-la (*p* < 0.05). (**b**) Resolubilization degree *RD* of the precipitated and washed α-la and of resolubilized α-la samples after pH adjustment and CaCl_2_ addition. Data are given as mean ± SD, *n* = 2. * Significantly different than precipitated α-la (*p* < 0.05).

As a scale of precipitate dissociation of the α-la refolding batches, the OD_550_ and the *RD* determined by RP-HPLC quantification were measured after pH adjustment and CaCl_2_ addition. Resolubilized α-la samples in the pH range of 6.0–10.0 in steps of 0.5 pH-units were investigated. The results of OD_550_ are shown in Figure 1a, the results of *RD* determination are presented in Figure 1b.

All samples with pH values in the acidic range showed high turbidity, which indicates a predominance of precipitates instead of single protein molecules. A remarkable jump in turbidity decrease was observed between pH 7.5 and pH of 8.0. With a further increase of the pH value the nominal OD_550_ was reduced slightly further. Based on pairwise statistical comparison, the refolding pH values 8.5 to 10.0 did not significantly differ from each other. However, the turbidity is only an indicator, but not a direct measurement of the extent nor of the quality of α-la’s refolding. Additionally, the *RD* was determined based on RP-HPLC quantification of insoluble and soluble α-la (Figure 1b). In the precipitated and washed α-la only 3.5% of the α-la was soluble. With a pH increase to pH 6.0 only a slight improvement of solubility to 8.9% was observed, though not being significantly different from the precipitated references. The biggest step in resolubilization was obtained between pH 6.5 and pH 7.0. The *RD* of the samples refolded at pH 8.0 to 10.0 lay between 91.9–93.2% and were not significantly different from each other.

Generally, the *RD* results support the OD_550_ measurements from a qualitative perspective. The *RD* results may suggest a more extensive resolubilization at a lower pH value. However, this shift may also be related to the sample preparation method that includes dilution. Both methods are in good accordance with results from literature. For example, Bramaud et al. [24] also reported an effective resolubilization of α-la starts at pH 8.0.

As described in the introduction, the α-la precipitates are mainly stabilized by hydrophobic interactions. The alkaline pH decreases the hydrophobic interactions, while the importance of the electrostatic forces increases and lead to repulsion of the molecules [36,37]. It is required to singularize the α-la molecules to make the calcium-binding site accessible for the subsequently added calcium.

In other experiments, the amount of added calcium was varied between 1 to 10-fold stoichiometric amount in respect to the number of α-la molecules. However, results indicated that the calcium concentration was not the main influencing factor in resolubilization. The results with 1 to 2 times calcium showed slightly inferior resolubilization results compared to the higher concentrations. The results with 3 to 10-fold calcium concentration were equal within a 95% confidential interval. The results are in agreement with literature, stating that addition of excess CaCl_2_ only affects the transition temperature but does not change the protein substructures involved in the refolding [38].

### 3.2. Investigation of Thermal Stability

**Figure 2:**
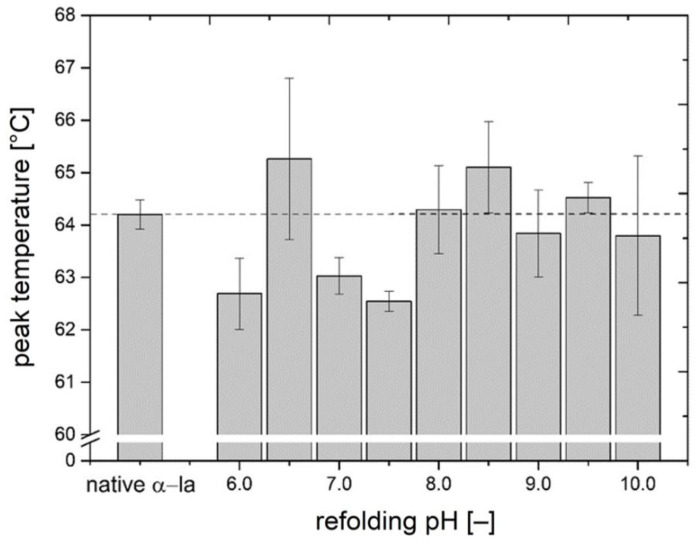
Peak denaturation temperatures of α-la samples that were refolded at pH values between 6.0–10.0 in comparison with untreated native reference α-la (left), determined by DSC analysis. Data are given as mean ± SD, *n* = 2. All refolded batches were significantly different from native α-la (*p* < 0.05).

The denaturation temperature was determined by DSC and provides information about the proteins’ thermal stability, which strongly correlates with the tertiary structure of the protein. An identical or similar denaturation temperature suggests that two proteins follow a similar road map in unfolding and that the same binding types, such as thiol bridges and hydrogen bonds, are accessible and dissociate at the same threshold temperature. The peak denaturation temperature of native calcium-bound α-la was determined at 64.2 °C, which is indicated by the dashed line across the diagram in Figure 2. The peak denaturation temperature of the native standard is in good accordance with values from literature [39,40].

The refolded α-la samples showed peak denaturation temperatures between 62.5–65.3 °C. The samples being refolded at pH 6.0, 7.0, and 7.5 showed a denaturation temperature of more than 1 °C below the one of the native reference. Other refolded batches, such as pH 6.5, 8.5, and 9.5, demonstrated higher denaturation peak temperatures than the reference. The best match in denaturation peak temperature was achieved at a refolding pH of 8.0 with a peak temperature of 64.3 °C. However, the difference in peak temperature of all refolded batches compared to the native reference was proven to be significant (*p* < 0.05). This is a first indication that the structural conformation of the refolded batches differs from the native state.

The samples with pH values from 8.5–10.0 had relatively high standard deviations. One possible explanation is the presence of partially irreversibly denatured α-la or of protein fragments that might occur as a consequence of mechanical separation forces. Another reason could be that the refolded α-la samples may contain several different conformations in one sample. Time-resolved IR studies suggest that the thermally unfolded apo-α-la likely initially transitions to a molten globule state, followed by either parallel or sequential folding pathways to resume the native holo-α-la state [41]. Though, a correct refolding to native state remains a challenging task, especially, when the protein was kept in a thermally unfolded state for a longer time and was further exposed to mechanical stress through stirring and centrifugation. Literature data indicate that the binding of Ca^2+^ to the calcium-binding site of α-la initiates a folding nucleus at the interface between the α- and β-domain, which appears to be a crucial step to trigger the correct re-organization of the native structure [42]. The two domains are held together by the disulfide bridges between Cys_73_ and Cys_91_ and between Cys_61_ and Cys_77_. The total of four disulfide bonds are considered crucial for the stability of α-la, even when transitioned to apo-form. The disulfide bridges in α-la can resist temperatures higher than 90 °C, when α-la is heated alone or no reactive sulfhydryl groups are present, such as β-lg exposes at temperature above 60 °C [43,44]. Therefore, it is very likely that the four disulfide bonds remained intact throughout the processing.

With the peak denaturation temperature all being higher than 62 °C, it is expected that the refolded α-la samples have a Ca^+^ bound. The apo-form is described to unfold already at temperatures as low as low as 10 to 30 °C [4]. Though, the results fail to clearly indicate whether the native calcium-binding site is restored or not.

### 3.3. Investigation of Hydrophobicity

**Figure 3:**
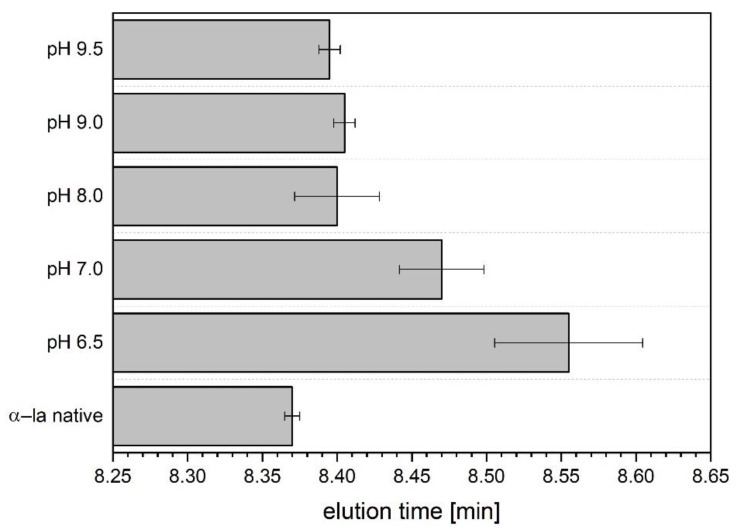
Elution times in RP-HPLC analysis of native reference and refolded α-la samples at different pH values. Data are given as mean ± SD, *n* = 2. All refolded batches were significantly different from native α-la (*p* < 0.05).

The RP-HPLC separates proteins according to their surface hydrophobicity, i.e., the hydrophobic stationary phase has a stronger affinity for hydrophobic compounds, which is the reason why more hydrophilic molecules are eluted first. With the increasing acetonitrile gradient, the hydrophobic interactions between the stationary phase and the molecule are gradually reduced, and thus hydrophobic molecules elute at a later point of time. This separation principle was used to investigate if the refolded α-la samples show differences in hydrophobicity, which would relate to a deviation in the tertiary structure. Figure 3 shows that the elution time of the native reference α-la was 8.37 min. The α-la samples being refolded at a pH of 6.5 and 7.0 had a distinct later elution time, which means that these refolded samples have a higher hydrophobicity compared to the native reference. The closest match in elution time compared to the native reference is achieved with the sample that was refolded at pH 8.0 with a time of 8.38 min.

Statistical analysis resulted in a significant difference in elution time of all refolded samples compared to the native reference.

Globular proteins locate most of their hydrophobic amino acid residues in the inner core [45], and so does α-la [46]. With the precipitation procedure used in this study, the α-la releases its calcium ion and unfolds to a certain extent, thus exposing the inner hydrophobic parts [47]. All refolded batches of α-la had a later elution time, implying a higher surface hydrophobicity. These results indicate that there are still inner hydrophobic residues exposed that are usually not accessible.

### 3.4. Analysis of Secondary Structure

FTIR spectrometry is an established method to determine a structural ‘fingerprint’ of a protein by providing insights in its secondary structure. The second-derivative spectra of native α-la sample was used to determine the approximate position and number of peaks in the Amid I band. Each of these peaks can be assigned to certain secondary structure motive of proteins. The assignment of structural motifs was performed based on results of Carbonaro and Nucara [35] and is listed in the Supplementary Materials Section as Table S1.

**Figure 4:**
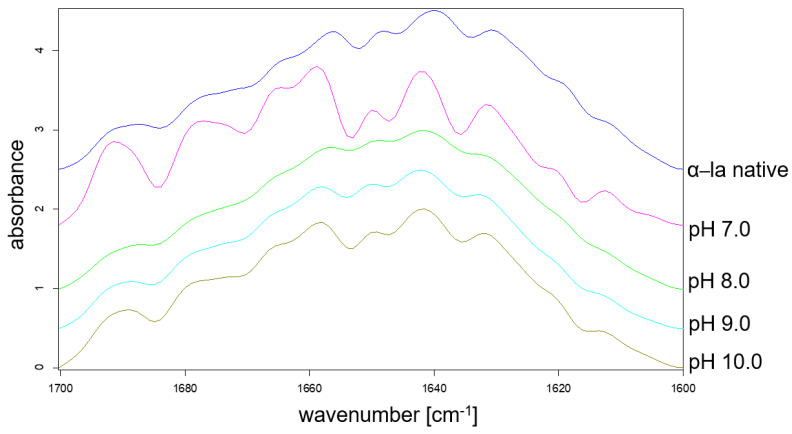
FTIR spectrum of the Amid I band of native α-la is compared to the batches that were resolubilized at pH values between 7.0 and 10.0.

In Figure 4, the Amid I band spectrum of native α-la is compared to the spectra of the samples that were refolded at different pH values. The spectrum of the pH 7.0 refolded batch shows distinct differences, especially in the region of 1700–1680 cm^−1^, as well as 1620–1600 cm^−1^, which are assigned to β-sheet and β-turn structures (Table S1). Such an extensive formation of β-structures is characteristic for protein aggregation [48]. This supports the assumption that a resolubilization pH of 7.0 is not sufficient to induce a proper dissociation of the α-la molecules from precipitated state, which is supported by the results from Figure 1. The spectra of samples refolded at pH 8.0–10.0 show a certain resemblance to the spectrum of the native α-la, at least from a qualitatively perspective. Nevertheless, the peak heights still show discrepancies to the native reference.

**Figure 5:**
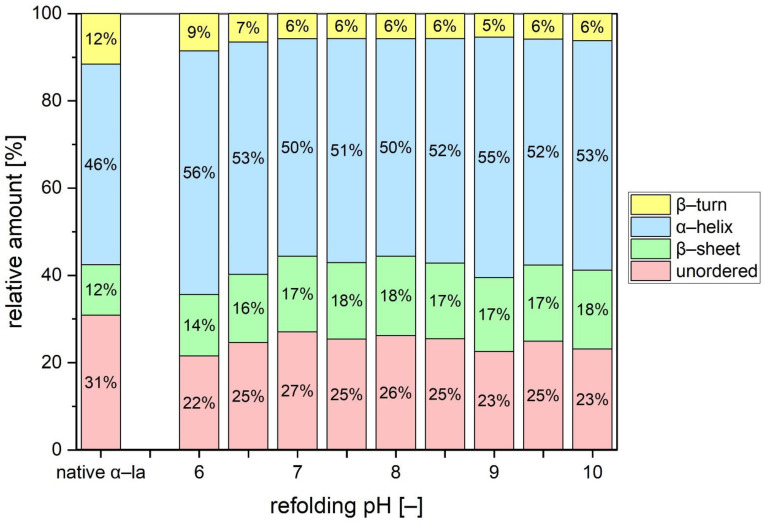
The relative amounts of secondary structural motives calculated based on FTIR curve areas displayed for the refolded batches and the native α-la sample. Data are given as mean, *n* = 2. All refolded batches were significantly different from native α-la (*p* < 0.05).

A Gaussian fitting was performed with the spectra from Figure 4 considering that nine peaks were determined previously. The relative area under each of the Gaussian curves was calculated and assigned to a respective secondary structural motive, as listed in Table S1. Figure 5 shows the relative amounts of β-turns, α-helices, β-sheets, and unordered structure for the refolded batches as well as for the native reference.

The distribution of the structural elements clearly shows that for none of the refolded samples, the identical conformation from the native state was entirely restored. Statistical analysis proved that the difference to the native reference was significant. All refolded batches comprise a lower amount of β-turns and unordered structure, whereas the relative amount of α-helices and β-sheets had increased.

Boye [49] investigated FTIR spectra of α-la samples at different pH values that were heated and subsequently cooled. At both, acid and alkaline pH, the cooled samples showed a loss of β-turns compared to native reference. Furthermore, they reported on an increased β-sheet formation in the samples heated and cooled under acidic conditions. This was attributed to intensified intermolecular interactions due to increased hydrophobicity upon unfolding, which finally resulted in the formation of intermolecular β-sheets. It was suggested that this formation of intermolecular β-sheets may indicate a (irreversible) denaturation of the protein or the formation of α-la dimers. Other studies suggest that non-native β-sheet formation may transiently appear, especially, when the refolding is slow [50]. In fact, the refolding to the native apo-structure in absence of calcium was reported to be slower than when calcium is present [51]. As described in the materials section, the calcium was added after the pH adjustment, which might affect the ability to refold properly.

Intermolecular interactions are likely the reason why a complete restoration of the native state was not achieved. Kuhlman et al. [52] demonstrated that the native C-helix is crucial for formation of the calcium-binding site and promotes a proper connection of the two domains of α-la. In case the C-helix is sterically hindered, e.g., by intermolecular crosslinks, the normal connection of the α- and β-domain would be impaired. The resulting cleft would result in inner hydrophobic amino acids still being exposed to some extent, which is supported by the previously presented elution time results.

Generally, refolding to the native conformation from a thermally unfolded state requires a correct formation of the β-sheets domain, the calcium binding site and the numerous loop and turn structures, as well as the restoration of a stable tertiary structure. Major changes in the environment, however, which could be caused by different pH values, temperature or solvent conditions, can affect the folding of the β-sheet domain and the structure of the loops in the molten globule [53]. Furthermore, apo-α-la is not only more susceptible to thermal treatment, but also to hydrostatic and mechanic stress compared to its holo-form [18,54]. It is likely that the applied conditions, such as heating to 50 °C for several hours and the mechanical stress through stirring and centrifugation are the root cause for the deviating conformation after refolding.

## 4. Conclusions

The aim of this study was to determine to which extent the original and native structure of α-la—after having been exposed to chemical, thermal, and mechanical stress—can be re-established at alkaline pH and calcium addition. The overall outcome was that under none of the investigated refolding conditions, the exact native structure was fully restored. Among the different investigated refolding batches, the use of pH of 8.0 and a three-fold stoichiometric amount of calcium showed the highest resemblance to the native reference. Under these resolubilization conditions, the denaturation temperature was only 0.1 °C higher compared to the native α-la, although the resemblance was not statistically significant. In the analysis of RP-HPLC elution time, the pH 8.0-refolded α-la was also closest to the native α-la by a difference of less than 1 s. Even though the absolute shift in elution time seems to be comparably low, the difference was significant different from the native reference. A comparison of the full Amid I band spectra of native and resolubilized samples suggests an overall resemblance of the peak profile, but some respective secondary motifs have distinctly changed by the precipitation and resolubilization procedures. In conclusion, all analytical methods confirmed that, within a confidence interval of 95%, the native structure of α-la was not completely re-established by the presented resolubilization method. However, the degree of deviation across all analytical methods applied was not very large.

Generally, a refolding of thermally unfolded apo-α-la to a native holo-state appears to be possible, as it was demonstrated by various studies previously. However, these studies investigated the refolding process under isolated lab conditions, without any further stress on the proteins. In contrast to those works, this study examined the applicability of the refolding potential under real life conditions, i.e., in a technologically relevant environment. Although the four disulfide bridges seem to stay intact and although it appears as if the structurally decisive calcium ion between two domains of the native α-la was in fact re-integrated, the precipitation method obviously has remaining effects on the conformation of the refolded α-la samples. Overall, our results demonstrate that characteristic structural motifs were reorganized sustainably as a result of chemical, thermal and mechanical treatment of the protein. Therefore, the resolubilization results could be termed ‘restructuring’ rather than renaturation.

We see the main application field of the restructured α-la in functional and/or fortified foods, such as α-la-enriched infant formula. For this type of application, the key property is the amino acid composition, which is generally not affected by the procedure used in this study. If and to which extent the ability of cation binding was impaired should be verified in further experiments. The results of the denaturation temperature determined by DSC is, however, a strong indicator that the re-integration of the calcium into the protein’s structure has taken place.

The applied precipitation conditions were chosen based on previous studies, ascribing highest precipitation yield and best separation results to this method. In this case, the high yield and good separation efficiency is achieved at the expense of a minor restructuring of α-la. Given that this separation concept does not only yield β-lg unaffected by the processing conditions applied, but also α-la in a soluble state, there is no loss in the whey protein main components, i.e., no low value side stream is produced. This way, the main whey proteins α-la and β-lg can both be obtained in a still functional state both from a nutritional and technological perspective.

## Data Availability

Data sharing does not apply to this article.

## Supplementary Materials

The following are available online at https://www.mdpi.com/article/10.3390/foods10092231/s1, Table S1: Assignment of secondary structural motifs to the peak positions obtained from FTIR spectrum analysis.
