# Supplementary material for: Molecular Analytical Assessment of Thermally Precipitated α-Lactalbumin after Resolubilization
Source: Foods. 2021 Sep 20;10(9):2231. doi: 10.3390/foods10092231 (PMC8465387; doi:10.3390/foods10092231)
Supplement: Supplementary file 1 [file foods-10-02231-s001.zip › foods-1362578-supplementary.pdf]

Table S1. Assignment of secondary structural motifs to the peak positions obtained from FTIR spectrum analysis.

Peak Position [cm^{-1}]	Secondary Structure
1693	β-sheet
1680	β-turn
1666	α-helix
1658	α-helix
1650	α-helix
1643	unordered
1632	α-helix
1620	β-sheet
1612	β-sheet
